# Humanized Mouse Models for the Study of Human Malaria Parasite Biology, Pathogenesis, and Immunity

**DOI:** 10.3389/fimmu.2018.00807

**Published:** 2018-04-19

**Authors:** Nana K. Minkah, Carola Schafer, Stefan H. I. Kappe

**Affiliations:** ^1^Center for Infectious Disease Research, Seattle, WA, United States; ^2^Department of Global Health, University of Washington, Seattle, WA, United States

**Keywords:** *Plasmodium falciparum*, humanized mouse models, FRG human hepatocyte, human immune system mice, malaria vaccines

## Abstract

Malaria parasite infection continues to inflict extensive morbidity and mortality in resource-poor countries. The insufficiently understood parasite biology, continuously evolving drug resistance and the lack of an effective vaccine necessitate intensive research on human malaria parasites that can inform the development of new intervention tools. Humanized mouse models have been greatly improved over the last decade and enable the direct study of human malaria parasites *in vivo* in the laboratory. Nevertheless, no small animal model developed so far is capable of maintaining the complete life cycle of *Plasmodium* parasites that infect humans. The ultimate goal is to develop humanized mouse systems in which a *Plasmodium* infection closely reproduces all stages of a parasite infection in humans, including pre-erythrocytic infection, blood stage infection and its associated pathology, transmission as well as the human immune response to infection. Here, we discuss current humanized mouse models and the future directions that should be taken to develop next-generation models for human malaria parasite research.

## Malaria: Disease Burden, Parasite Life Cycle, Natural Immunity, and the Development of a Malaria Vaccine

Malaria, a disease caused by protozoan *Plasmodium* parasites, causes more than 200 million clinical cases annually and is responsible for more than 400,000 deaths each year, mainly in children under the age of 5 and pregnant women living in the resource-poor countries of sub-Saharan Africa. In humans, the majority of malaria infections are caused by *Plasmodium falciparum* and *Plasmo-dium vivax*. In more temperate regions of the world, socioeconomic development, vector control programs, and the use of antimalarial chemotherapeutics have driven successful malaria elimination. However, declines in malaria infections have been slowest in tropical, resource-poor countries with a high malaria burden necessitating the development of new effective antimalaria therapeutics and vaccines that will prevent infection, disease, and onward transmission.

**Figure 1:**
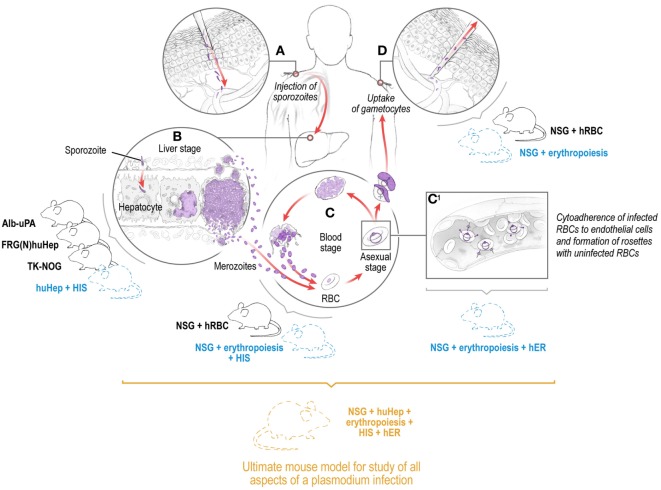
Depiction of the *Plasmodium* life cycle in humans showing the skin, liver, and blood stages with the corresponding existing (solid lines) and future (dashed lines) humanized mouse systems to model each of the individual stages and eventually the full *Plasmodium* life cycle including pathology and transmission. Infection is initiated when a female *Anopheles* mosquito injects saliva-containing sporozoites into the skin. Sporozoites traverse dermal cells and gain access to the blood **(A)**. The highly motile sporozoites transit to the liver where each sporozoite infects a single hepatocyte **(B)**. One to two weeks after hepatocyte invasion, merozoites exit the liver and begin a 48-h cycle of red blood cell (RBC) invasion, replication, RBC rupture, and new merozoite release **(C)**. During RBC infection, the parasite expresses variant surface antigens on the surface of the infected red blood cell, which interacts with human endothelial receptors (hER), thus mediating the binding of infected RBCs to the microvascular endothelium of various organs **(C1)**. A small number of blood-stage parasites differentiate into sexual gametocytes, which are taken up by mosquitoes in blood meals to continue the transmission to new human hosts **(D)**.

Transmission of malaria parasites to the mammalian host begins with the deposition of the infectious, motile sporozoite stages into the skin via the bite of infected mosquitoes (1–3). Sporozoites initially traverse multiple cell types in the skin in search of capillaries to gain access to the bloodstream within which they are transported to the liver (3). Each sporozoite infects a hepatocyte, then transforms into a liver stage that undergoes growth, genome replication, and differentiation into tens of thousands of red blood cell (RBC) infectious exo-erythrocytic merozoites. In humans, merozoites are released from the liver into the bloodstream 7–10 days after initial transmission where they infect RBCs, replicate within, and are released to undergo continuous cycles of infection, replication, and release, allowing parasite numbers to reach billions within weeks (Figure 1). In *P. vivax* infection, a subset of sporozoites form persistent liver stages (hypnozoites) that activate at later time points to cause relapsing blood stage (BS) infections (4, 5). All symptoms associated with malaria are caused by BS infection, and this is in large part due to the massive destruction of RBCs but also the sequestration of infected RBCs in the microvasculature (6). This sequestration can occur in a tissue-specific manner, leading, for example, to cerebral malaria pathology through sequestration of infected RBCs in the brain or pregnancy associated malaria due to sequestration of infected RBCs in the placenta. Uptake of parasite sexual forms in a blood meal leads to infection of the mosquito, sporogonic development, and colonization of salivary glands by sporozoites, which ensures transmission to new human hosts (7, 8) (Figure 1).

Repeated *Plasmodium* infection does not result in complete immunity, rendering populations in endemic regions continuously susceptible to infection, malaria-associated morbidity and mortality as well as transmission. A fully protective malaria vaccine has yet to be developed (9). Moreover, evolution of parasite resistance to frontline antimalarial drugs necessitates continuous research and development of next-generation antimalarials (10). These efforts require robust experimental systems that accurately model human malaria parasite biology, immunology, and pathogenesis. Given the tropism of the human *Plasmodium* species for human hepatocytes (huHeps) and RBCs, researchers have relied on *in vitro* infection models for human parasites to query malaria biology and identify targets of intervention. In this review, we will discuss how blood, tissue, and immune system-humanized mouse models can provide novel avenues to examine human malaria parasites. Humanized mice and their past use in malaria research have been reviewed recently (11, 12). Therefore, in this review, we will focus more on critical research gaps in our understanding of human malaria parasite biology, pathology, and immunology that might be addressed in humanized mouse models and improvements to these models that are needed to achieve this. We will also reflect on the role of next-generation multi-compartment-humanized mice in the modeling of the complete *Plasmodium* life cycle in physiologically relevant human cells and tissues.

## Pre-Erythrocytic (PE) *Plasmodium* Infection: Biology of Transmission - The Skin and Liver Stages

The sporozoite and liver stages comprise the PE stage of infection. Unlike the BSs, the PE stages are asymptomatic, are small in numbers during natural infection, and are not as antigenically variant. These characteristics render them extremely attractive targets for malaria intervention (13). Dissimilar to the BSs, the PE stages of *P. falciparum* cannot be easily generated in the laboratory and although some aspects of human infection can be modeled *in vitro*, these systems have limitations. Therefore, most of our knowledge on PE biology has been derived from studies of rodent malaria parasites, which were originally isolated from wild African rodents and subsequently adapted to laboratory mice (14–16). For transmission research, the biology of skin infection between different rodent parasite species (*Plasmodium yoelii* and *Plasmodium berghei*) made it attractive to speculate that parasite behavior in the skin should be similar in *Plasmodium* species that infected humans. However, the recent identification of the development of exo-erythrocytic merozoites in the skin of *P. berghei*-infected mice (17) but not in *P. yoelii*-infected mice (18) identified one point of divergence even within these two closely allied parasite species. Thus, whether the development of skin exo-erythrocytic merozoites occurs in human *Plasmodium* parasites remains an open question, as preventive therapeutics targeted to the liver stage might be ineffective in the skin. Given that this skin stage of infection can only be adequately modeled within the three-dimensional architecture of the skin tissue, recent advances in the engraftment of human skin into immunodeficient mice (19) represent an exciting opportunity to examine interaction of human parasites with human skin components *in vivo* and an opportunity to explore the occurrence and relevance of “skin exo-erythrocytic merozoites” in *Plasmodium* parasites that infect humans.

Each sporozoite that reaches the liver invades a single hepatocyte, transforms into a trophic stage, and then commences liver stage development (also called exo-erythrocytic development). Traditional rodent malaria models have been critical to the iden-tification of host hepatocyte surface factors necessary for sporozoite invasion (7, 20). Recently, human liver-chimeric mice have been employed to examine the contribution of these invasion factors in human malaria parasite liver infection (21). The efficient engraftment of huHeps into mice is dependent on an environment of severe immunodeficiency to limit huHep rejection coupled with the elimination of mouse hepatocytes to provide the huHep a niche in the liver parenchyma. Three different mouse models have been utilized for huHep engraftment and have been used to assess liver stage infection by *Plasmodium* parasites infecting humans. The SCID Alb-uPA model expresses the toxic urokinase plasminogen activator (uPA) under the control of an albumin promoter in the livers of the highly immunodeficient *S*evere *C*ombined *I*mmune *D*eficiency mice (SCID Alb-uPA). Upon engraftment with huHeps, these mice become susceptible to infection with *P. falciparum* sporozoites and support complete liver stage development including the release of exo-erythrocytic merozoites that egress and invade human RBCs (huRBCs) *ex vivo* (22, 23). An alternate to the induction of hepatotoxicity by uPA transgene expression is genetic ablation of *fumarylacetoacetate hydrolase* (FAH) in mice resulting in acute liver failure which can be rescued by administration of the drug, 2-(2-nitro-4-fluoromethlbenzoyl)-1,-3-cyclohexanedione (NTBC). Crossing these FAH^−/−^ mice onto the severely immunocompromised C57BL/6 Rag2^−/−^IL2rγ^−/−^ mouse generated FAH^−/−^Rag2^−/−^IL2rg^−/−^ (FRG) mice. These mice have also been backcrossed onto the non-obese diabetic (NOD) background (FRGN), which additionally renders them hospitable to transplantation with CD34^+^ hematopoietic stem cells (HSCs) (24). Using NTBC cycling during engraftment, these mice can exhibit over 90% engraftment with huHeps (FRG huHep) (25), are susceptible to infection with both *P. falciparum* and *P. vivax* sporozoites (26, 27) and support full liver stage development, including the release of exo-erythrocytic merozoites capable of invading huRBCs that were infused into the mice. When infected with *P. vivax*, FRG huHep mice also harbor non-replicating hypnozoites (27). More recently, the TK-NOG (NOD/Shi-scid/IL2rg^−/−^) mouse has been developed as yet another mo-del for *P. falciparum* PE infection (28). These mice express the herpes simplex virus thymidine kinase transgene under the control of the albumin promoter on the NOD SCID IL2rγ^−/−^ background. Destruction of mouse hepatocytes is achieved by treatment with ganciclovir, allowing repopulation of the liver with huHeps. TK-NOG mice support *P. falciparum* and *Plasmodium ovale* sporozoite infection and liver stage development (28).

## Anti-PE *Plasmodium* Immunity and Vaccine Development: From Traditional Mouse Models to Human Clinical Trial

The pronounced human host cell tropism of malaria parasites that infect humans precludes infection of the traditional immunology workhorse, the in-bred mouse. Thus, the rodent malaria parasites, *P. yoelii* and *P. berghei* have been extensively utilized because they allow a careful examination of PE immunity. Perhaps the greatest contribution of the rodent malaria models is in the examination of immunity and protection against an infectious sporozoite challenge after vaccination with whole, attenuated sporozoites. Attenuation was originally generated by gamma irradiation (29) but can now be achieved by genetic engineering with the precise removal of genes from the parasite genome (30, 31) or by treatment of an infectious sporozoite immunization with drugs that prevent BS infection (32, 33). Over the last decade, studies utilizing whole sporozoite infection of mice have identified roles for humoral immunity (3, 34–37) and both peripheral and tissue-resident memory CD8 T cells in the protective response to immunization (13, 38–41). Previously thought to be immunologically silent, liver stage *Plasmodium* infection also induces an innate immune response (42–45). The pathways by which this innate immune response is induced and the influence it has on the ensuing adaptive immune response are areas of active investigation.

Although traditional rodent models of PE infection have been useful, differences in rodent and human *Plasmodium* species (46) compounded with significant divergence in rodent and human hosts presents significant implications for the extrapolation of results achieved with the former to the latter, particularly with regard to host–parasite interactions and immunity. Also, while the liver stages of rodent *Plasmodium* species develop fully within 2–3 days, the human *Plasmodium* parasites undergo 7–10 days of liver stage development before exiting the liver to infect erythrocytes. In addition, none of the rodent parasites form persistent liver stages that could model those found in *P. vivax* infection, affirming that PE biology in the rodent is not an ideal model for human malaria infection. Human clinical trials have identified robust induction of both humoral and cellular immune responses after whole sporozoite immunization yet unequivocal identifi-cation of correlates of protection from these studies has proven to be challenging. To reconcile divergent observations, functional *in vitro* assays have been developed such as the examination of immune sera and its inhibitory activity on infection of cultured hepatoma cell lines with sporozoites (47–49). Yet, *in vitro* cultured cells do not accurately model the complex architecture of the liver tissue, rendering them only partially physiologically relevant as infection assays. In addition, tissue-resident memory T cells, which have been shown to be critical in the control of liver stages in rodent malaria infection, do not recirculate into the blood stream (40), impeding the examination of their contribution to PE immunity after *P. falciparum* immunization of humans. Peripheral T cell populations that correlate with protection have been identified in rodent *Plasmodium* models (50). However, no *ex vivo* assays exist to robustly quantify CD8 T cell killing of hepatocytes or the augmentation of humoral and cellular immune responses by CD4 T cells. Thus, many questions remain regarding the relevance of these cell populations for protection in humans.

## Humanized Mice to Model PE Immunity

Given the lack of robust *ex vivo* and *in vitro* assays to functionally examine human immune responses to *Plasmodium* infection and immunization with candidate vaccines, the identification of true correlates of protection will require the development of animal models that better mimic human *Plasmodium* infection. Traditionally, this role has been occupied by non-human primates (NHP) where *Plasmodium* infection better mirrors observations made in humans. Numerous NHP species support infection by *Plasmodium* species that occur in simians and some NHP support direct infection with *P. falciparum* and *P. vivax* (51). Moreover, the immune response in NHP is similar to humans and tissue-resident immune populations can be sampled to query their importance to vaccine-engendered protection (52). However, NHP systems are still not a perfect model for human *Plasmodium* infection, and in addition to ethical and financial barriers, logistical concerns regarding housing requirements for NHPs have curtailed their use in malaria research. Thus, the development of immunodeficient mice that have been engrafted with functional human immune cells and human tissues will enable the examination of infection in human cells and tissues in the context of human immune responses *in vivo* within a small animal model.

Human liver-chimeric mice can play important roles to study functionality of human immune responses. They might serve as an important and cheaper alternative to NHP models in the final down-selection of antibody-based vaccine antigens. For example, we and others have utilized these mice to demonstrate that the passive transfer of human monoclonal antibodies or polyclonal sera from humans immunized with whole *P. falciparum* sporozoites, blocks liver infection (53–55). Of note, mosquito bite challenge of FRG huHep mice after passive transfer allowed superior discrimination of antibody-mediated protection as compared to established *in vitro* assays. However, given the absence of an adaptive immune response in these mice, active immunization experiments cannot be carried out.

To analyze intrinsic human immune responses, human immune system (HIS) mice have been developed that enable the direct analysis of the HIS responses *in vivo* after infection with human pathogens or after vaccination. HIS mice are generated by the transplantation of human CD34^+^ cells containing HSCs into immunodeficient NOG or NSG mice, to model relatively normal human immune responses (56). Huang and colleagues have recently pioneered the generation of HIS mice that possess functional human CD4, human CD8, and human B cell responses (57, 58) for the study of PE immunity. To generate humanized mice with a competent human humoral response (referred to as HIS-CD4/B mice), immunocompromised NSG mice were transduced with recombinant adeno-associated virus (AAV) vectors encoding human HLA class II (HLA DR1 or HLA DR4) and a cocktail of human cytokines followed by engraftment with human CD34^+^ cells (58). To examine the ability of these HIS-CD4/B mice to mount a protective humoral response, HIS-CD4/B mice were first immunized with recombinant *P. falciparum* circumsporozoite protein (CSP) and then subsequently challenged with a transgenic *P. berghei* parasite encoding the repeat regions of *P. falciparum* CSP. Immunized HIS-CD4/B mice exhibited high titers of circulating *P. falciparum* CSP antibodies and showed reduced parasite liver burden after challenge as compared with naïve controls (58).

Given the importance of CD8 T cells in PE-engendered protection in rodent models of *Plasmodium* infection, Huang and colleagues also generated a humanized CD8 T cell mice referred to as HIS-CD8 (57) by transducing NSG mice with AAV vectors encoding functional HLA-A*0201 and a cocktail of human cytokines. Immunization of HIS-CD8 mice with AAV vectors bearing *P. falciparum* CSP resulted in an induction of HLA-restricted human CD8 T cells. These immunized HIS-CD8 mice greatly reduced parasite burden in the liver after challenge with transgenic *P. berghei* parasites encoding full length *P. falciparum* CSP (59). Importantly, *in vivo* depletion of human CD8 T cells completely abolished the reduction in liver burden in immunized HIS-CD8 mice (60).

These HIS studies, however, only support challenge with transgenic rodent malaria parasites expressing selected *P. falciparum* proteins. Thus, there is a need to combine humanized liver-chimeric mice with the HIS models to generate dual-chimeric mice (HIS huHep) susceptible to human *Plasmodium* liver infection and capable of driving functional human immune responses. However, such a model will not yet completely replicate human immunity because although liver-chimeric mice can be repopulated with high levels of huHeps, their liver sinusoidal endothelial cells, Kupffer cells, hepatic stellate cells, and cells of the myeloid lineage all remain of mouse origin. In rodent malaria systems, CD8^+^ dendritic cells have been shown to be critical to the generation of an effective immune response after whole sporozoite immunization (61, 62). In addition, in *P. berghei*-infected mice, IFNAR expression on myeloid cells is critical for the propagation of the innate immune response to whole parasite infection (42). What roles these cells play in the effective PE immune response to human whole sporozoite immunization remains unknown. Finally, given the importance of liver-resident memory CD8 T cells in PE immunity (38–40), it will be important to examine whether HIS-CD8 mice recapitulate the critical roles of tissue-resident cells observed in rodent malaria studies.

## Humanized Mice to Model Malaria BS Infection

Although *P. falciparum* BSs can be cultured *in vitro*, a small animal model of human BS malaria would offer great advantages, as it would allow the preclinical testing of drugs and vaccine candidates in an *in vivo* setting against the human pathogen. The liver-chimeric mice described above support liver infection and liver stage-to-BS transition after injection of target huRBCs (27, 63). The presence of huRBCs on the day of exo-erythrocytic merozoite egress from the liver leads to a short period of low parasitemia, and these parasites can then be removed and maintained in huRBC culture. However, BS infection cannot be maintained in the mice as huRBCs are rapidly cleared. Fortunately, different immune-modulation protocols combined with daily injections of huRBCs can support high engraftment levels and promote a continuous *P. falciparum* BS infection in NSG mice (64). These mice show sequestration of the parasite in bone marrow and spleen, suggesting it might resemble the behavior of the parasite in humans. One drawback is that in this study the mice were directly infected with BS parasites, as they are not human liver-chimeric. Moreover, as *P. falciparum* gametocytes take 10–14 days to mature, this is the time span the infected RBC has to be maintained to allow transmission back to the mosquito. If this is achieved, the model might enable the study of transmission in an *in vivo* setting and might allow the testing of transmission blocking drugs and vaccines before moving on to clinical trials. Our laboratory has recently developed a robust protocol to engraft and maintain huRBCs in human liver-chimeric mice to better assess the efficacy of transmission blocking small molecules, antibodies and vaccines (65). Another promising application for combined huHep/huRBC mice is the preclinical evaluation of safety of attenuated *P. falciparum* whole sporozoite vaccine candidates, allowing for exquisite sensitivity in detecting potential breakthrough infection into the blood before testing of new attenuated strains in human trials (66). Furthermore, combined huHep/huRBC mice have been successfully used for the recovery of recombinant parasite progeny from *P. falciparum* genetic crosses. Previously, such genetic crosses had to be carried out in splenectomized chimpanzees, but it was recently reported that recombinant progeny can also be recovered from FRG huHep mice that had been injected with huRBCs at the time point of merozoite egress from the liver (67). The option to perform genetic crosses in a small animal model provides a robust avenue for forward genetics research and a new avenue to determine the underlying traits of *P. falciparum* drug resistance and other phenotypes of clinical importance.

Whereas *P. falciparum* BSs can be easily cultured *in vitro*, all efforts to establish a long-term *in vitro* culture for *P. vivax* have so far met with limited success. Therefore, research on this widespread parasite would especially benefit from a small animal infection model. The distinct feature of *P. vivax* BS parasites is the strong preference for CD71^+^ reticulocytes (68). These are highly immature erythrocytes that are mainly found in the bone marrow. Consequently, high amounts of *P. vivax* ring stage infected cells are also found in the bone marrow (69). To establish a humanized mouse model that will propagate *P. vivax* BS infection, mice will have to be engrafted with these rare cells. Reticulocytes account for only 0.5–2% of peripheral blood, of which only a very small fraction are CD71^+^. Higher numbers of CD71^+^ cells are found in umbilical cord blood (UCB) and enriched reticulocytes from UCB have been successfully used for in *P. vivax in vitro* BS invasion assays (70). It remains to be determined whether the amount of target cells in mice engrafted with the enriched reticulocyte fraction from UCB would be sufficient to propagate a *P. vivax* infection. One avenue to achieve high numbers of CD71^+^ reticulocytes is to differentiate human HSCs into erythroid precursor cells. These cells should closely resemble *P. vivax* target cells and therefore might support a *P. vivax* BS infection *in vivo*. Encouragingly, infection of FRG huHep mice with *P. vivax* sporozoites leads to development of liver stages that undergo full schizogony and release exo-erythrocytic merozoites. When provided with reticulocyte target cells at the time points of release, infection of and development of asexual BSs was observed (27). FRG huHep mice also support hypnozoite persistence, giving hope that a combined liver and blood model for *P. vivax* could one day enable the routine study of relapsing infection (27).

The repeated injection of huRBCs, combined with different immunomodulatory protocols, is a cumbersome process, necessitating an experienced researcher and often leading to losses of mice. An elegant alternative to repeated huRBC reconstitution would be a mouse that intrinsically sustains human erythropoiesis after HSC transplantation. Especially regarding the cell tropism of *P. vivax* described above, a model in which human erythropoiesis takes place in the bone marrow would hopefully provide a setting in which *P. vivax* BSs can develop. Unfortunately, in the humanized mouse models published to date, human erythropoiesis is severely impaired (71). The injection of human cytokines important for human erythropoiesis only leads to a small increase in huRBCs in the periphery (72). A mouse in which human CD34^+^ HSC transplantation leads to robust human erythropoiesis combined with an HIS, and which in addition harbors a human-chimeric liver, would ultimately enable the production of reproducible data on the developmental life cycle of human malaria parasites. Therefore, it is a high priority goal for malaria research to develop a humanized mouse model, which intrinsically promotes human erythropoiesis and can then be combined with one of the human liver-chimeric models described above. Such an advanced humanized mouse model would open up completely unexplored avenues of research. For example, despite its obvious importance, there is an enormous lack of knowledge in the area of *Plasmodium* coinfections with other pathogens. Malaria and HIV/AIDS especially have a wide geographical overlap, particularly in sub-Saharan Africa, and epidemiological studies have shown cross-contribution to each other’s pathogenicity (73), potential antimalarial treatment failure in HIV^+^ patients (74) as well as potential drug–drug interactions (75). Very little is known about the underlying molecular mechanisms of these observations, as we lack a model system to investigate them. Currently, the questions of drug–drug interactions or vaccine safety in co-infected individuals can solely be addressed in clinical trials, as carried out previously for the malaria vaccine candidate RTS, S (76). However, the advanced mouse model described above, with a human-chimeric liver, human erythropoiesis and HIS, could potentially fill this gap.

## BS Malaria Pathology in Humanized Mice

The question of whether a humanized mouse model might enable the study of *P. falciparum* malaria-associated pathophysiology remains largely unexplored. The pathology of severe malaria is mainly determined by adhesion interactions between infected erythrocytes and human endothelial cells. These adhesion interactions lead to the sequestration of infected erythrocytes in the microvasculature, which benefit the parasite by avoiding clearance in the spleen. Unfortunately, this sequestration also leads to vascular occlusion and inflammation, which are important contributors to severe malaria pathology. Three forms of adhesive interactions have been described: the cytoadherence of infected erythrocytes to endothelial cells, formation of rosettes with uninfected erythrocytes and platelet-mediated clumping of infected cells (77). These interactions are mediated by the *P. falciparum* erythrocyte membrane protein 1 (PfEMP1) family, variant antigens expressed on the surface of infected RBCs that interact with multiple host receptors, including ICAM1, CD36, E-selectin, and endothelial protein C receptor (EPCR) (78). There are approximately 60 different variants of PfEMP1, which are encoded by genes of the *var* family but only expressed one at a time. Depending on which *var* gene is expressed, the parasite modifies the antigenic properties of infected erythrocytes, which allows it to evade the host immune system but also changes the binding specificity for host receptors. *Var* gene switching is currently under extensive investigation and a small animal model allowing the controlled *in vivo* evaluation of this phenomenon would be of great benefit to this important field of research.

The most lethal complication of a *P. falciparum* infection is cerebral malaria. It has been speculated that the targeting of different receptors via the expression of different PfEMP1 variants leads to tissue-specific sequestration of the parasites. In the brain microvasculature, the EPCR has been shown to play an important role in the sequestration of parasites (79). To date, we lack any knowledge of whether infected RBCs sequester in the brain of *P. falciparum*-infected humanized mice. It is thus highly relevant to investigate whether the described pathologies of human malaria are recapitulated in any of the humanized mouse models described above. If high huRBC reconstitution and high parasitemia can be achieved in humanized mice it is likely that rosetting of infected erythrocytes will take place in a mouse model. Whether PfEMP1 molecules on infected erythrocytes interact with mouse receptors on endothelial cells remains an unanswered question, however. If this is not the case, a mouse model must be developed in which human receptors are expressed on mouse endothelial cells. If this could be achieved it would open up new possibilities for exploring the potential of anti-adhesion drugs or antibodies as novel malaria therapies.

## BS Malaria Immunity in Humanized Mice

Animal models of malaria continue to provide important insights into the immune response to *Plasmodium* BS infection in general. However, the development of novel immunotherapeutic strategies against BS parasites requires a thorough investigation of the human immune response, particularly to *P. falciparum* BS infection. Our understanding of the human immune response to early BS infection, vaccination, and natural infection has been gleaned from longitudinal studies on subjects enrolled in controlled human malaria infection trials (80–82). Together with the rodent *Plasmodium* studies, two immunological processes critical for the control of BS malaria infection have been identified, namely, the inflammatory response from innate immune sensing of *Plasmodium* infections and the humoral response. *Plasmodium* pathogen-associated molecular patterns engage pattern recognition receptors on innate immune cells and trigger an inflammatory response critical for early parasite control (83, 84). However, this inflammatory response can also be pathogenic to the host. Indeed, *Plasmodium*-engendered type I IFN signaling impairs dendritic cell function in *Plasmodium chabaudi* (85) and *P. berghei*-infected mice (86) and promotes the production of the immunosuppressive cytokine, IL-10 by Tr1 cells in a human CHMI trial (87). Antibodies have long been known to play a central role in BS malaria immunity (88). Yet, over half a century later, our understanding of the parasite proteins that induce protective antibody responses, the mechanisms of *Plasmodium*-engendered humoral protection and why protective antibodies only develop after years of repeated exposure is finally maturing. Mounting evidence in rodent models and correlative data in humans from endemic regions have established that *Plasmodium* evades humoral immunity through dysregulation of CD4^+^ T cell (89) and B cell dysfunction (90–95). Although the mechanisms behind the roles of inflammation on dendritic cell function and B cell dysfunction have been well studied in rodent models of malaria, a mouse model with a humanized immune system will be critical to confirming whether the mechanisms outlined in the rodent malaria models apply to infection with the human *P. falciparum* parasite. However, as described in preceding sections, the existing humanized mouse models will not accurately mimic a complete HIS as they still contain murine antigen presenting cells, or other murine myeloid cells. Mice that retain human antigen presentation and human myeloid cell function will need to be developed to measure these effects.

## Conclusion

Conventional mouse model infections with rodent malaria parasites have critically contributed to our understanding of malaria parasite biology, pathogenesis, and immunology and have been important in malaria vaccine and drug discovery. However, differences between the genomes of the human-infective and rodent-infective *Plasmodium* species as well as significant divergence between mouse and human biology might preclude facile application of knowledge gleaned from traditional rodent systems to the design of effective interventions in humans. Humanized mouse models have emerged as a critical link between traditional rodent models and humans. huHep mice will enable better examination of the factors critical for hepatocyte infection and liver stage development and the understanding of liver stage-directed, infection-preventing interventions. HIS mice are poised to greatly accelerate our understanding of the immune response to human *Plasmodium* parasites and vaccine candidates as the data gleaned from these studies will more closely represent the immune response in humans. However, current iterations of these HIS mice retain mouse myeloid compartments likely influencing antigen presentation and immune cell residency. Next-generation HIS mice for malaria research will likely require humanization of the liver, bone marrow, lymphoid compartments, and human erythrocytes. BLT (bone marrow, liver, and thymus) mice, where human fetal thymus and liver tissues as well as autologous HSCs are engrafted into the same mouse represent the most complete humanized mouse system to date (96). Combining this with huHep mice would generate the triple-humanized mouse sorely needed for the study of human malaria parasite infection and immunology. However, the high costs and technical demands of such a system will likely preclude it from being widely employed. Ultimately, future humanized mouse models for *Plasmodium* research will utilize the transplantation of CD34^+^ HSC cells to develop robust human immune and erythropoietic compartments and hepatocyte transplantation to ensure human liver chimerism. Ideally, this mouse will in addition harbor human receptors on endothelial cells so that the pathobiology of malaria BS infection can also be modeled. As it will take great effort to create such a complete and complex mouse model, a continued iterative cycle of basic parasitological discovery and immunology in rodent malaria models combined with studies in currently available, albeit imperfect huHep, HIS, and huRBC mice will further increase the predictive value of animal models for human clinical intervention.

## Author Contributions

NM and CS prepared the original drafts of this manuscript with subsequent revision by SK.

## Conflict of Interest Statement

The authors declare that the research was conducted in the absence of any commercial or financial relationships that could be construed as a potential conflict of interest.
